# The impact of renal artery stenting on therapeutic aims

**DOI:** 10.1038/s41371-022-00785-8

**Published:** 2022-12-16

**Authors:** Ben Edgar, Robert Pearson, Ram Kasthuri, Keith Gillis, Colin Geddes, Maggie Rostron, Adrian Brady, Keith Hussey, Giles Roditi, Christian Delles, Linsay McCallum, Patrick Mark, David Kingsmore

**Affiliations:** 1 Glasgow Renal and Transplant Unit, Queen Elizabeth University Hospital, Glasgow, UK; 2 Department of Radiology, Queen Elizabeth University Hospital, Glasgow, UK; 3 School of Cardiovascular & Metabolic Health, University of Glasgow, Glasgow, UK; 4 Department of Vascular Surgery, Queen Elizabeth University Hospital, Glasgow, UK

**Keywords:** Renovascular hypertension, Atherosclerosis, Renovascular hypertension

## Abstract

Renal artery stenosis manifests as poorly-controlled hypertension, impaired renal function or pulmonary oedema, therefore the success of treatment is dependent on indication. This study aims to determine the outcomes of patients undergoing renal artery stenting (RASt) based on therapeutic aim compared to criteria used in the largest randomised trial. Retrospective case-note review of patients undergoing RASt between 2008–2021 (*n* = 74). The cohort was stratified by indication for intervention (renal dysfunction, hypertension, pulmonary oedema) and criteria employed in the CORAL trial, with outcomes and adverse consequences reported. Intervention for hypertension achieved significant reduction in systolic blood pressure and antihypertensive agents at 1 year (median 43 mmHg, 1 drug), without detrimental impact on renal function. Intervention for renal dysfunction reduced serum creatinine by a median 124 μmol/L, sustained after 6 months. Intervention for pulmonary oedema was universally successful with significant reduction in SBP and serum creatinine sustained at 1 year. Patients who would have been excluded from the CORAL trial achieved greater reduction in serum creatinine than patients meeting the inclusion criteria, with equivalent blood pressure reduction. There were 2 procedure-related mortalities and 5 procedural complications requiring further intervention. 5 patients had reduction in renal function following intervention and 7 failed to achieve the intended therapeutic benefit. Renal artery stenting is effective in treating the indication for which it has been performed. Previous trials may have underestimated the clinical benefits by analysis of a heterogenous population undergoing a procedure rather than considering the indication, and excluding patients who would maximally benefit.

## Introduction

Renal artery stenosis (RAS) is an anatomical categorisation of several disease processes that result in reduced renal perfusion [1]. The impact is two-fold: directly—through reduced renal perfusion resulting in reduced function and ultimately ischaemic atrophy; and indirectly—through activation of the renin-angiotensin-aldosterone and sympathetic nervous systems leading to systemic hypertension, cardiovascular complications and ultimately, end-organ damage including glomerulosclerosis [2]. The most common causes of RAS are atherosclerosis and fibromuscular dysplasia (FMD). Atherosclerotic RAS is not uncommon and may be present on imaging in up to 50% of patients with coronary artery disease [3]. In contrast, the incidence of pathologically impaired renal perfusion leading to clinical conditions is much less clear with the clinical presentation influenced by speed of onset and the type/severity of organ dysfunction.

Given the variable presentation of symptomatic RAS, there may be several therapeutic aims of treating RAS either acutely (reducing blood pressure, improving renal function, treating acute cardio-renal syndromes) or in the longer term (preventing hypertensive end-organ damage and ischaemic renal atrophy). The current treatment of clinically relevant RAS consists primarily of medical optimisation with anti-hypertensive agents [4]. Endovascular treatment may be undertaken with angioplasty alone (usually only in patients with FMD) or with stenting (RASt) in atherosclerotic disease, where ostial calcification makes recoil and restenosis more likely if angioplasty is performed in isolation. It is possible that amongst populations with a heavy atherosclerotic burden, a significant number of people could merit intervention, but the role of RASt is uncertain as two large randomised clinical trials (RCTs) failed to show a significant benefit to either renal or cardiovascular outcomes [5, 6].

The two trials differed in rationale—inclusion to the Angioplasty and Stenting for Renal Artery Lesions (ASTRAL) trial being clinical equipoise in the presence of both anatomical atherosclerotic stenosis in at least one renal artery that was suitable for endovascular revascularization, whereas the Cardiovascular Outcomes in Renal Atherosclerotic Lesions (CORAL) trial used clinical and anatomical inclusion criteria, although these were modified mid-recruitment. The subsequent interpretation that all revascularisation is of limited benefit may be an over-interpretation of these RCTs, which were designed to study a very defined group in which there was perceived equipoise. ‘High-risk’ subgroups of patients that may have been most likely to benefit were excluded, and patients in whom there was likely to be little or no benefit were included [7–9]. Consequently generalising the results of these exclusive RCTs may have inappropriately led to the conclusion that RASt is hazardous and has no benefit [10, 11]. Perhaps most importantly, the outcome by which these RCTs measured success was not based on the rationale for performing the procedure with no differentiation between hypertension, renal dysfunction or indeed acute cardio-renal syndromes.

In contrast to ASTRAL and CORAL, the STAR trial [12] did focus on rationale, examining the impact of stenting on renal impairment in patients with atherosclerotic RAS. The results were inconclusive, demonstrating some efficacy to stenting but also a considerable complication rate. Again, however, high-risk patients with refractory hypertension were excluded, and no data is presented on the rate of decline in renal function. The findings are disputed by a prospective study by Reinhard et al. [13], which reports favourable outcomes of renal artery stenting in high-risk patients including those with rapidly declining renal function.

The aim of this study was therefore to evaluate the role of RASt in a contemporary practice based on the therapeutic aims, with a comparison to the set criteria used in the CORAL trial.

## Materials and methods

A retrospective analysis was performed of all patients who had undergone RASt in a defined geographical area in the West of Scotland (population 2.4 million) across three centres between 2008 and 2021. Patients with a diagnosis of atherosclerotic RAS were identified through a word-search of the radiology information system in which all imaging and interventions are reported. From this, patients in whom RASt was performed in a native renal artery outwith a clinical trial were identified, and the records reviewed using both the electronic patient records and the prospectively maintained Scottish Electronic Renal Patient Record (SERPR, Vitalpulse).

Data extracted included patient demographics and the indication for RASt (hypertension; renal impairment—acute or chronic; pulmonary oedema). Blood pressure measurements were calculated by taking the mean value of 3 readings using an automated oscillometric device under office conditions. Acute renal dysfunction was defined as a rise in serum creatinine concentration by 25% of its baseline figure within the 6 months preceding intervention. Chronic renal dysfunction was defined by stable sub-normal function that had not deteriorated by 25% in the preceding 6 months. Patients on dialysis at the time of stenting were excluded from calculations analysing changes in serum creatinine. Procedural details and outcomes (including blood pressure, medication, and renal function) were recorded. The outcomes of intervention were described from the time of intervention (immediate post-intervention, and long-term by the last date of recorded data). Complications were also defined by time: peri-procedural (<24 h), early (24 h to 30 days); late (more than 30 days following intervention). Deterioration in renal function was defined by a rise of more than 25% of the serum creatinine concentration from pre-intervention levels and maintained for more than 60 days, where it could not be attributed to other causes.

Failure to achieve therapeutic aim was defined by the indication: for hypertension, failure to reduce blood pressure or anti-hypertensive medication if blood pressure not changed; for renal impairment, failure to reduce creatinine or recovery of function if on dialysis at the time of procedure; and for flash pulmonary oedema failure to avoid recurrence.

The cohort was also stratified using the criteria employed in the CORAL trial. A CORAL exclusion group [5] was identified [chronic kidney disease from causes other than ischaemic nephropathy (*n* = 2); serum creatinine >354 µmol/L [4.0 mg/dL] (*n* = 12); target kidney <7 cm (*n* = −0); lesions that could not be treated with a single stent (*n* = 6); allergy to medications in protocol (*n* = 0); multiple renal arteries (*n* = 2); and artery <3.5 mm in diameter (*n* = 0)] with the comparator being patients suitable for entry into the trial – referred to as the CORAL inclusion group.

### Statistical analysis

Data was collated using Microsoft Excel (Version 16.53 © Microsoft 2021). Statistical analysis was performed using RStudio (Version 1.4.1717 © 2009–2021 RStudio, PBC. Means were compared by Paired T-test, Welch Two-Sample T-test or Mann-Whitney U Test as appropriate.

## Results

**Fig. 1:**
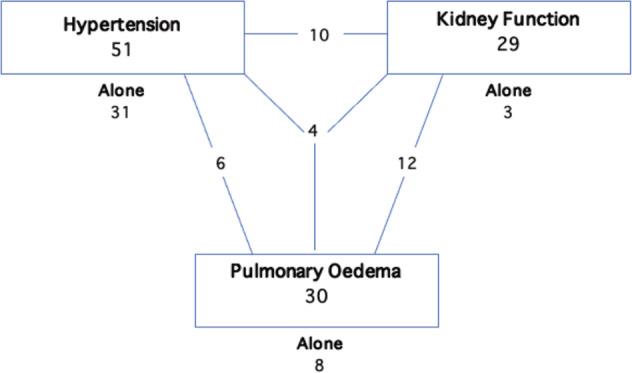
Indication for renal artery intervention. Multiple indications expressed by adjoining lines.

Over the 13-year period 74 patients had RASt with a mean age of 61 ± 16 years, an equal sex distribution (49% male) with a mean follow-up of 68.8 months (median 1832 days, range 870–3132, total 154,422 days). Hypertension was the most common indication and was commonly treated in isolation (31 out of 51 treated), whereas the other indications were rarely seen in isolation (pulmonary oedema: 8/30, impaired renal function: 3/29; Fig. 1). Baseline-demographic characteristics of the study population are shown in Table 1.

**Table 1:** Baseline demographic characteristics of the study population.

	Overall	Indication			CORAL	
		HTN	RF	PO	Included	Excluded
*N*	74	51	29	30	52	22
Age, mean (sd), *years*	63 (15)	58 (16)	69 (10)	68 (10)	62 (16)	59 (17)
% Male	47	45	48	43	43	59
Vascular history						
Any vascular disease, *n* (%)	41 (55)	24 (47)	21 (72)	23 (76)	31 (59)	10 (45)
Diabetes, *n* (%)	12 (16)	6 (12)	6 (21)	7 (23)	8 (15)	4 (18)
Cerebrovascular disease, *n* (%)	16 (22)	10 (20)	8 (28)	9 (30)	12 (23)	4 (18)
Ischaemic Heart Disease, *n* (%)	31 (42)	16 (31)	17 (59)	18 (60)	23 (44)	8 (36)
Peripheral Arterial Disease, *n* (%)	17 (23)	12 (24)	9 (31)	10 (33)	14 (27)	3 (14)
Blood pressure						
Systolic blood pressure, mean (sd), mmHg	187 (30)	195 (27)	180 (27)	178 (28)	187 (30)	184 (32)
Diastolic blood pressure, mean (sd), mmHg	89 (19)	94 (20)	84 (15)	82 (13)	89 (21)	89 (12)
Antihypertensive medications						
Prescribed medications, mean (sd)	3 (1)	4 (1)	3 (1)	3 (1)	3 (1)	3 (1)
Diuretic, *n* (%)	32 (43)	41 (80)	22 (75)	30 (100)	40 (76)	11 (50)
Doxazosin, *n* (%)	33 (45)	24 (47)	14 (48)	17 (57)	29 (55)	7 (31)
β-blocker, *n* (%)	42 (57)	28 (54)	15 (52)	20 (67)	34 (65)	12 (54)
ACE inhibitor, *n* (%)	20 (27)	16 (31)	3 (10)	4 (13)	18 (34)	6 (27)
Calcium-channel blocker, *n* (%)	42 (57)	30 (58)	17 (58)	19 (63)	39 (75)	11 (50)
Renal function						
Serum creatinine conc^n^, median (IQR), μmol/L	159 (193)	124 (136)	341 (175)	252 (182)	152 (148)	349 (342)
Estimated glomerular filtration rate, median (IQR), ml/min/1.73 m^2^	25 (35)	39 (35)	14 (10)	18.5 (15)	33 (35)	10 (7)

### Analysis by therapeutic aim

#### Hypertension (n = 51)

**Fig. 2:**
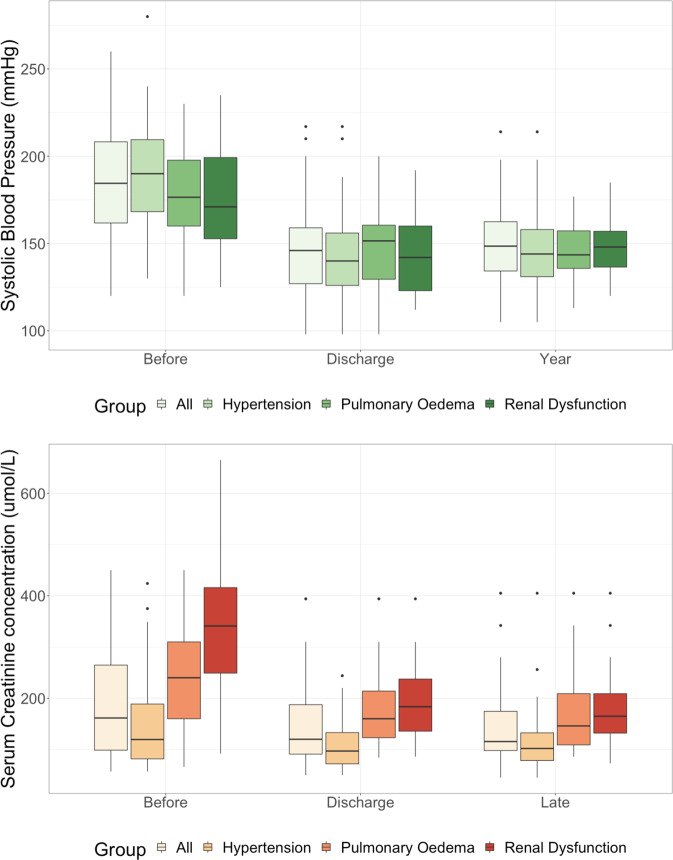
Change in blood pressure and serum creatinine following renal artery stent.

**Fig. 3:**
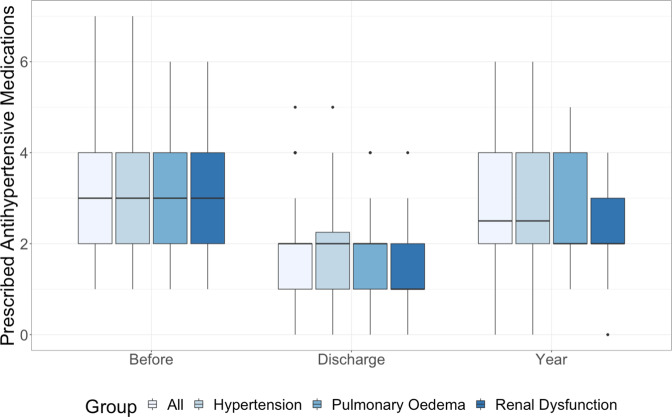
Changes in number of prescribed antihypertensive medications by indication.

**Fig. 4:**
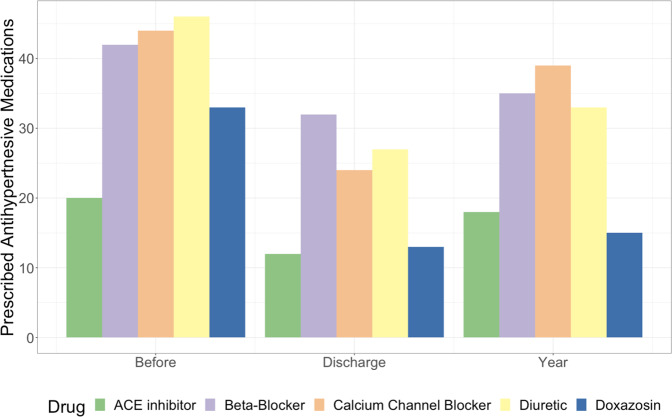
Changes in class of prescribed antihypertensive medication.

RASt led to a significant reduction in systolic blood pressure (a mean reduction from baseline of 42.4 mmHg at 1 year) and diastolic blood pressure (Figs. 2 & S1, Table 2 & S1) with the benefit of a reduction in the number of antihypertensive medications prescribed (median: 3 pre-RASt; 2 at discharge, and 2 at 1-year; Figs. 3 & 4). This was associated with a non-significant improvement in renal function (median reduction in serum creatinine of 22 μmol/l (IQR 1–63) at discharge and 24 μmol/l (IQR −4–55) at 6 months, *p* = 0.08 and 0.15). There was no difference by sex or age, and the benefit in reducing blood pressure was equally seen in patients with hypertension as the only indication and patients with hypertension with other indications.

**Table 2:** Systolic Blood Pressure measurements (mmHg) before and after renal artery stenting.

	Systolic Blood Pressure					
	Baseline		Discharge		1 Year	
	Mean	+/−	Mean	+/− *p*-value	Mean	+/− *p*-value
Overall *n* = 74	186.8	31.7	145.4	25.1 < 0.0001	149.3	23.5 < 0.0001
Indication						
Hypertension *n* = 51	194.6	30.6	143.7	25.8 < 0.0001	149.2	26.6 < 0.0001
Renal Dysfunction *n* = 29	179	28.5	144.2	23.2 < 0.0001	149.6	18.1 0.001
Pulmonary Oedema *n* = 30	179.5	28.5	148.1	23.7 < 0.0001	146.8	15.8 < 0.0001
CORAL						
Inclusion *n* = 52	186.5	29.1	144.9	26.8 < 0.0001	148	25.8 < 0.0001
Exclusion *n* = 22	183.8	32	146.8	21.3 < 0.0001	137.9	15.8 0.002

#### Renal function

Across the entire study group (*n* = 74) there was a significant reduction in creatinine on discharge following intervention (median reduction 37 [IQR 4–123] μmol/l, *p* = <  0.001) which was more marked at 6 months following intervention (median reduction 42 [IQR 1–141] μmol/l, *p* = 0.007; Table 3, Fig. 2). Reductions in median creatinine were accompanied by increases in median estimated glomerular filtration rate (eGFR) (Fig. S2, Appendix 1).

**Table 3:** Renal Function (Serum creatinine) before and after renal artery stenting. Median Serum creatinine in μmol/L. Patients on dialysis at time of stenting are excluded from this analysis.

	Serum creatinine					
	Baseline	IQR	Discharge	IQR *p*-value	1-year	IQR *p*-value
Overall *n* = 74	159	95-288	122	86-199 0.001	117	93-180 0.007
Renal dysfunction						
Acute *n* = 13	350	319–400	196	135–267 0.05	151	123–171 0.09
Chronic *n* = 16	318	243–441	204	153–280 0.09	205	149–327 0.09
Pulmonary Oedema *n* = 30	252	162–344	158	123–214 < 0.001	146	110–224 0.02
Hypertension *n* = 51	124	84–220	104	78–143 0.08	102	81–148 0.15
CORAL						
Inclusion *n* = 52	152	92–240	113	85–182 0.02	106	89–158 0.28
Exclusion *n* = 22	349	10s2–444	131	92–215 0.009	141	110–193 0.01

In patients with renal dysfunction as an indication (excluding those on haemodialysis), there was a significant reduction in serum creatinine immediately post-RASt with a further reduction at 6-months (median reduction: 124 [IQR: 15–216] μmol/l at discharge, *p* = 0.01 and 144 [IQR: 26–300] μmol/l at six months, *p* = 0.02). This reduction was seen in patients with both acute and chronic dysfunction (Table 3). The rate of improvement in function was more predictable and greater in the acute group (only one patient did not achieve a good response, Figs. S3 and S4, Appendix 1). Of 11 patients who had started haemodialysis prior to RASt, 10 regained function; 3 had presented with acute deterioration in renal function and 7 had acute deterioration on a background of chronic kidney disease, with 5 out of the 7 patients remaining dialysis free for the remainder of their life/to the end of follow-up. The mean dialysis-free time for the 5 patients who went on to require dialysis was 10.4 (±8.5) months. There was no difference by sex or age. The benefit on renal function was similar for renal dysfunction alone as well as patients with renal dysfunction in combination with hypertension or pulmonary oedema (*p* = 0.3).

#### Pulmonary Oedema

Patients in whom the indication was pulmonary oedema all had resolution of acute pulmonary oedema, but also gained significant improvements in creatinine and blood pressure following intervention. Serum creatinine fell by a median of 84 μmol/l [IQR 26-151] *p* = 0.0001 at discharge and 96 μmol/l [IQR 17–178] *p* = 0.02 at 6 months. Systolic blood pressure was reduced by 31 mmHg [SD 29.9] *p* = <0.001 at discharge and 33 mmHg [SD 29.1] *p* = <0.001 at 1 year. There was no difference by sex or age.

In the 12 months following intervention, 2 of the 30 patients (7%) had readmissions with recurrent pulmonary oedema, at 144 days and 335 days post-intervention.

#### Outcomes by CORAL criteria

**Fig. 5:**
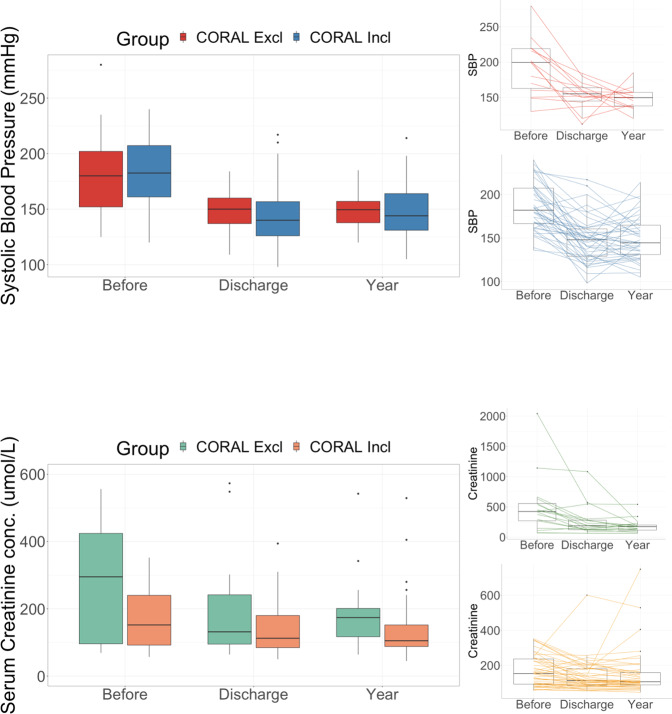
Change in blood pressure and serum creatinine following renal artery stent by CORAL criteria.

RASt led to significant reduction in blood pressure in patients that would have been both included or excluded from CORAL (Fig. 5). CORAL-excluded patients had a significantly greater reduction in serum creatinine than those who would have been included at discharge and at 6-months (218 [IQR 7–274] μmol/l versus. 27.2 [IQR 2–57] *p* = 0.02 and 208 [IQR 11–321] μmol/l versus 18.9 [IQR −3–59] μmol/l, *p* = 0.01 respectively). Both groups also had a significant reduction in systolic blood pressure that was sustained at 1 year (included group −37.5 mmHg [SD 27] *p* = <0.005 and excluded group - 44.9 mmHg [SD 45.7] *p* = 0.002). Those who would have been included had a mean reduction of 1 antihypertensive agent (SD 1.7, *p* = 0.03) at 1-year follow-up, whilst the exclusion group saw no significant change in the number of prescribed antihypertensives (0.1 SD 1.9, *p* = 0.8).

### Complications

#### Peri-procedural

There were 2 deaths, one following injury to an aberrant right hepatic artery, and one secondary to acute aortic dissection (mortality rate 2.7%). There were 12 (14%) peri-procedural complications: 8 puncture-site haematomas (3 requiring surgical haemostasis), 2 renal artery dissections (1 successfully managed with angioplasty and 1 leading to renal artery occlusion with infarction of the kidney), 1 thrombotic renal artery occlusion successfully managed using catheter-directed thrombolysis.

#### Early complications

There were 3 (5%) early complications: 2 renal artery thromboses, 1 of which was successfully treated by catheter-directed thrombolysis, the other managed with surgical revascularisation which, although successful in the medium-term, thrombosed at 5 months with haemodialysis ultimately required. 1 patient had a puncture-site pseudoaneurysm that required re-operation.

#### Long-term complications

There were 3 (4%) in-stent stenoses at 3, 5 and 21 months. Two were successfully treated with angioplasty and stent graft whilst the other was not amenable to intervention.

#### Deterioration in Renal Function

5 patients (7%) had a deterioration in renal function post-procedure of whom1 recovered normal renal function whilst the remaining 4 had a sustained increase in serum creatinine (median 75 [IQR 37–118] μmol/L *p* = 0.08). There was no association with indication and no long-term sequelae during the follow-up period.

#### Failure to achieve aim

7 patients (10%) failed to derive the intended benefit of intervention. 3 patients treated for hypertension (2 of these cases relating to contralateral RAS not amenable to percutaneous intervention) and 4 patients treated for renal dysfunction (all of whom had ongoing progressive chronic renal dysfunction, which continued at the same rate as before the intervention).

## Discussion

The aim of this study was to determine the impact of RASt on the therapeutic aim of the procedure, and relate the findings to the outcomes in patients in the CORAL trial. Using this analysis, we have demonstrated that RASt can successfully treat symptomatic renal artery stenosis, and that whilst almost one-half of the patients treated would not have been eligible for CORAL, a significant therapeutic beneficial impact was seen in all groups.

The results presented differ significantly from the CORAL and ASTRAL trials, which failed to show overall cardiovascular or renal benefits with the addition of RASt to best medical therapy. Partly this lack of benefit may be due to the rationale of the trials which analysed outcomes of a singular treatment, but based recruitment on a heterogenous population with differing therapeutic aims and potentially differing anticipated outcomes. This has consequence when balancing the anticipated benefits and risks for an individual patient. For example, when considering RASt for poorly controlled hypertension, the potential adverse impact of treatment (deterioration in renal function and mortality) must be balanced against the potential direct and indirect beneficial impact (a significant reduction in blood pressure and anti-hypertensive medication rather than persisting with polypharmacy and inadequately controlled blood pressure). In addition, the impact on blood pressure may not be directly relevant to patients with renal dysfunction or similarly the impact on renal function may not be as dramatic when hypertension is the indication.

Other limitations of these trials have been highlighted previously: [14, 15] ASTRAL randomised only patients in whom there was equipoise, excluding patients thought likely to benefit from intervention, and was designed to study patients with mild/moderate stenoses of uncertain clinical significance. The CORAL criteria changed during recruitment (with removal of the necessity for hypertension requiring 2 agents), had high recruitment losses (almost 20% of subjects withdrew, crossed-over or were lost to follow up), excluded patients with refractory hypertension or severe renal dysfunction, and included patients who were unlikely to benefit from intervention (normal blood pressure; mild stenoses). Almost 25% (1116 of 4375) of the patients excluded from CORAL did not have an explanation for exclusion. These limitations likely excluded those with most to gain from intervention [16, 17], and may have led to augmented risk from pharmacologically induced renal hypo-perfusion [8]. Furthermore, given that these RCTs recruited a population in which there was deemed clinical equipoise of intervention, the trial findings should not be generalised to populations where this is not the case, as this may lead to therapeutic nihilism. These suggestions appear to be supported in a subsequent re-analysis of CORAL data that demonstrated a significant benefit in 65% of participants who required 3 or more anti-hypertensives; where pre-RASt diastolic blood pressure >90 mmHg; and there was a requirement for Clonidine [18]. Recent reports have also highlighted beneficial outcomes in a cohort of patients at ‘high risk’ without intervention. In this ‘high-risk’ group (patients with pulmonary oedema, refractory hypertension or rapid deterioration of kidney function), RASt was beneficial in blood pressure control as well as improving kidney function [13, 19]. Analysis of such a group has been notably absent from the CORAL, ASTRAL and STAR trials [5, 6, 12].

There are limitations to this work. Data was collected retrospectively without knowledge of the outcomes of patients who were not offered intervention with inevitable selection bias. The rate of intervention was very low, with only highly selected intervention performed, reflecting the impact of the CORAL and ASTRAL trials. However, the degree of pre-operative antihypertensive medications in those with refractory hypertension suggests that a large proportion of patients were receiving optimal medical therapy. Therefore, the data presented should be considered as reassurance of the potential benefits of selective intervention, but does not help determine the wider role of conservative rather than interventional treatment of RAS. This would require a larger more inclusive RCT which given these results may be ethically problematic to justify.

In conclusion, this study has demonstrated that in selected patients, RASt can improve blood pressure control, reduce the number of prescribed antihypertensive agents and influence renal function. This must be balanced against the low rate of serious complications including mortality versus the potential complications of untreated symptomatic RAS. As these findings extend to a group of patients excluded from major trials, establishing of a prospective registry would allow evaluation of the effects of renal artery intervention in populations including those with refractory hypertension, flash pulmonary oedema and significant renal dysfunction and may better demonstrate the benefits of intervention.

## Summary

### What is known about this topic

- Atherosclerotic renal artery stenosis is a prominent cause of secondary hypertension. Treatment is primarily with anti-hypertensive medications, with vascular intervention in the form of angioplasty and/or stenting reserved for refractory cases.
- The role of renal artery stenting is uncertain as two large randomised controlled trials failed to identify significant cardiovascular or renal benefits in heterogeneous populations undergoing renal artery stenting.
- These trials, however, were designed to study a well-defined population in which high-risk patients who were most likely to benefit were excluded. Furthermore, they did not analyse outcomes based on the therapeutic aim of the procedure.

### What this study adds

- This study stratifies patients by the clinical indication for renal artery intervention; be it refractory hypertension, renal dysfunction, or an acute cardio-renal syndrome.

- The results of this study demonstrate that renal artery stenting is effective in treating the indication for which it is performed, and by comparing the study cohort to exclusion criteria used in the largest randomized trial, shows that these findings extend to a group of patients excluded from major trials.

### Supplementary information

Supplementary Figure and Table Legends

Figure S1

Figure S2

Figure S3

Figure S4

Table S1

## Data Availability

The datasets generated during and/or analysed during the current study are available from the corresponding author on reasonable request.
