# Correction: The impact of renal artery stenting on therapeutic aims

**DOI:** 10.1038/s41371-024-00893-7

**Published:** 2024-01-16

**Authors:** Ben Edgar, Robert Pearson, Ram Kasthuri, Keith Gillis, Colin Geddes, Maggie Rostron, Adrian Brady, Keith Hussey, Giles Roditi, Christian Delles, Linsay McCallum, Patrick Mark, David Kingsmore

**Affiliations:** 1 Glasgow Renal and Transplant Unit, Queen Elizabeth University Hospital, Glasgow, UK; 2 Department of Radiology, Queen Elizabeth University Hospital, Glasgow, UK; 3 School of Cardiovascular & Metabolic Health, University of Glasgow, Glasgow, UK; 4 Department of Vascular Surgery, Queen Elizabeth University Hospital, Glasgow, UK

**Keywords:** Renovascular hypertension, Atherosclerosis, Renovascular hypertension

Correction to: *Journal of Human Hypertension* 10.1038/s41371-022-00785-8, published online 16 December 2022

In this article authors Linsay McCallum and Patrick Mark have been added to the author list. The original article has been corrected.

