# Supplementary material for: The impact of renal artery stenting on therapeutic aims
Source: J Hum Hypertens. 2022 Dec 16;37(4):265–72. doi: 10.1038/s41371-022-00785-8 (PMC10063438; doi:10.1038/s41371-022-00785-8)
Supplement: Supplementary file 1 — Supplementary Figure and Table Legends [file 41371_2022_785_MOESM1_ESM.docx]

**Supplementary Figure Legends**

- Figure S1. Changes in Diastolic Blood Pressure following Renal Artery Stent
- Figure S2. Changes in Estimated Glomerular Filtration Rate following Renal Artery Stent. Laboratory results of ‘>60’ analysed as 70 ml/min/1.73m^2^.
- Figure S3. Change in Serum Creatinine following Renal Artery Stent by Renal Function Decline
- Figure S4. Pattern of Change in Serum Creatinine in Response to Renal Artery Stent by Renal Function Decline

**Supplementary Table Legends**

- Table S1: Diastolic Blood Pressure measurements (mmHg) before and after renal artery stenting
