# Supplementary figures and images for: The impact of renal artery stenting on therapeutic aims
Source: J Hum Hypertens. 2022 Dec 16;37(4):265–72. doi: 10.1038/s41371-022-00785-8 (PMC10063438; doi:10.1038/s41371-022-00785-8)

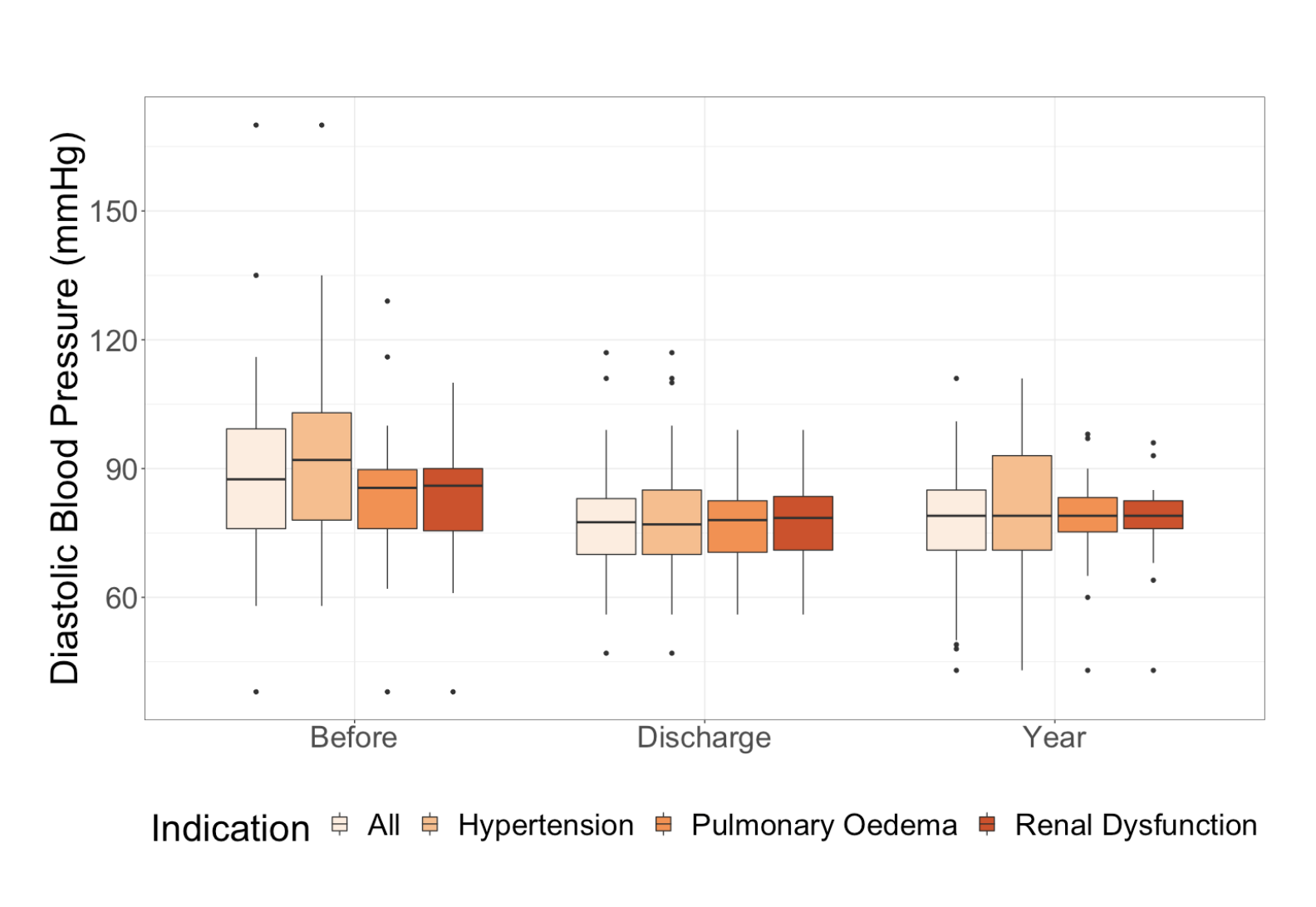

Supplement: Supplementary file 2 — Figure S1 [file 41371_2022_785_MOESM2_ESM.tif]

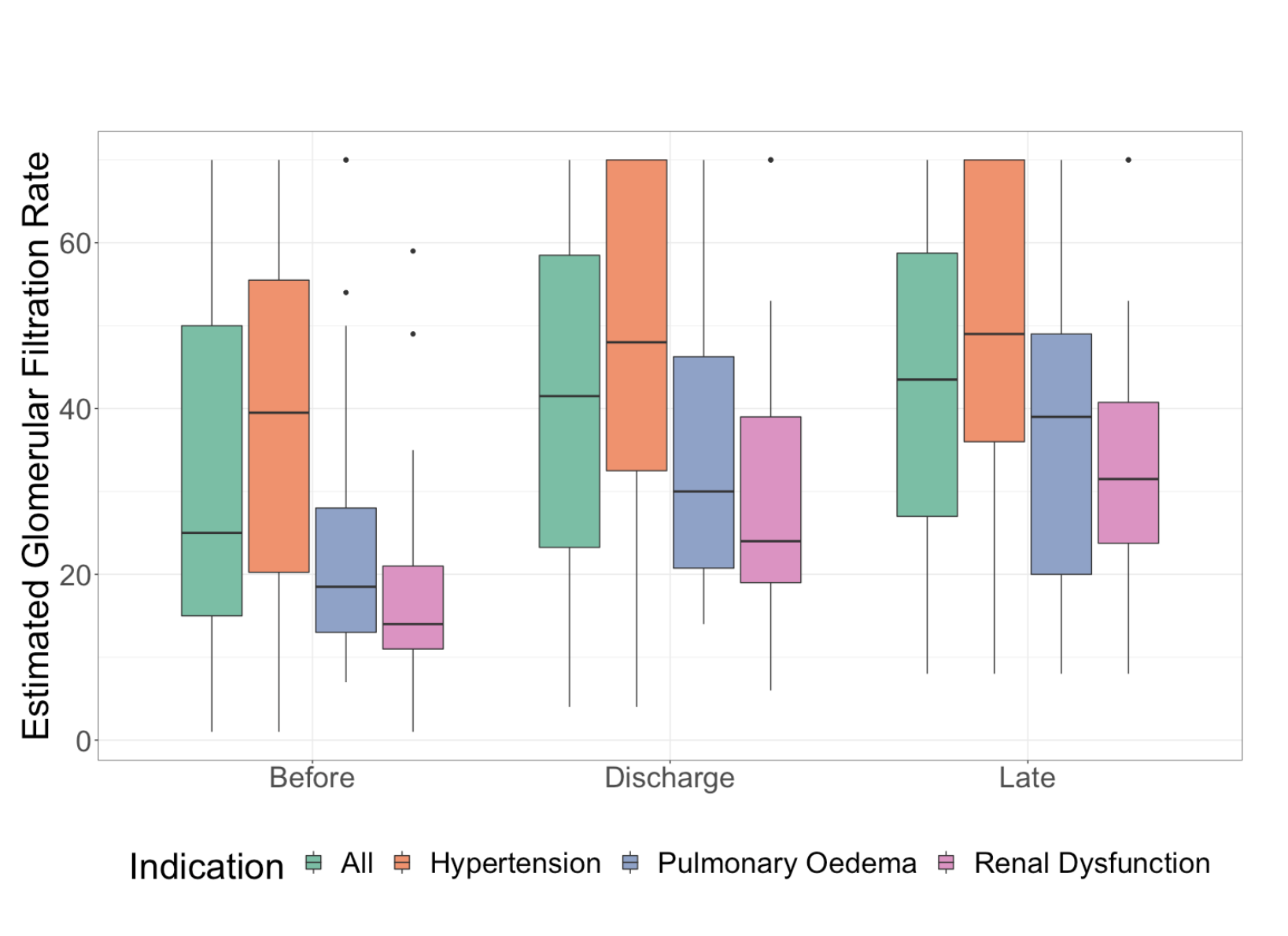

Supplement: Supplementary file 3 — Figure S2 [file 41371_2022_785_MOESM3_ESM.tif]

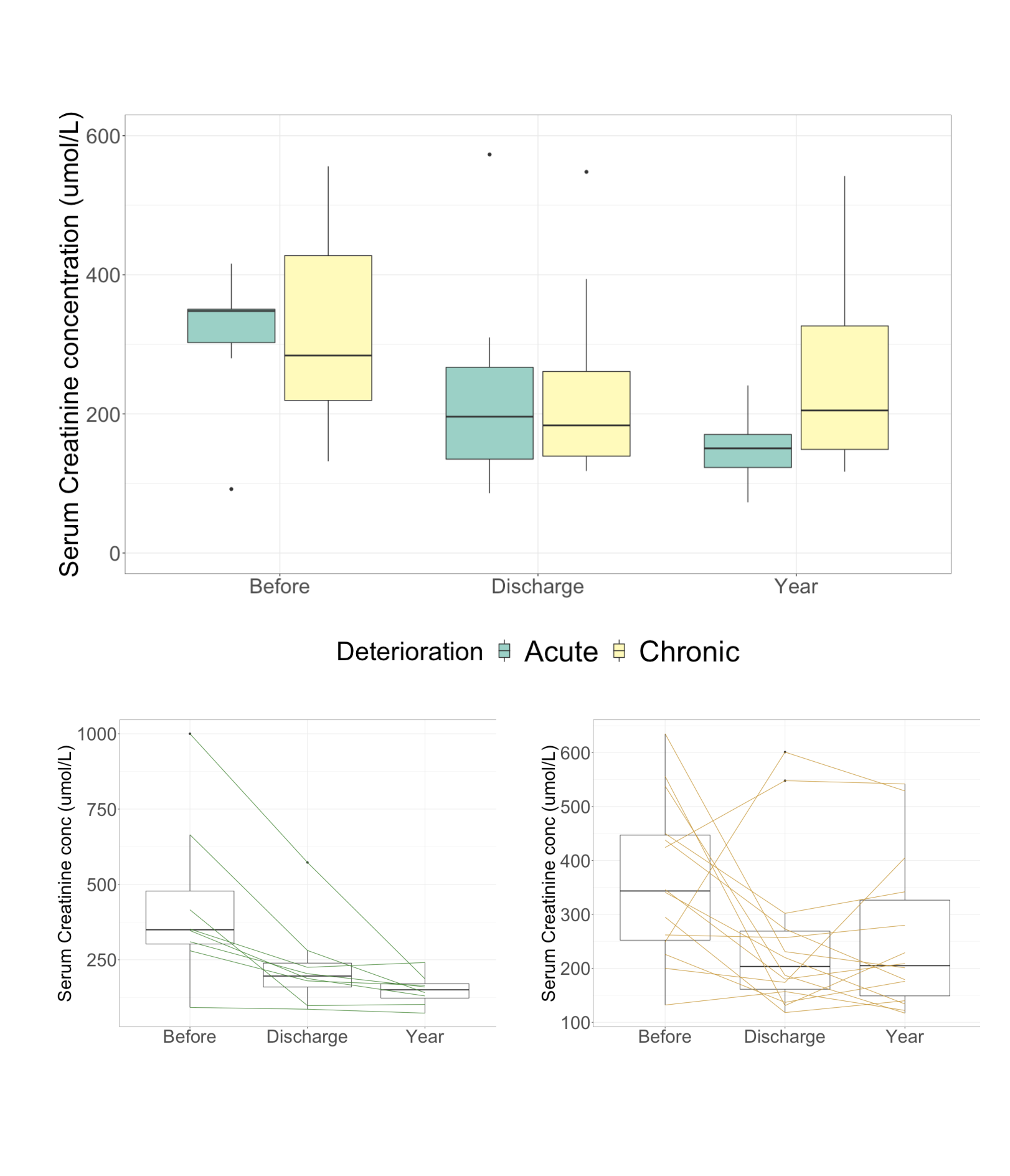

Supplement: Supplementary file 4 — Figure S3 [file 41371_2022_785_MOESM4_ESM.tif]

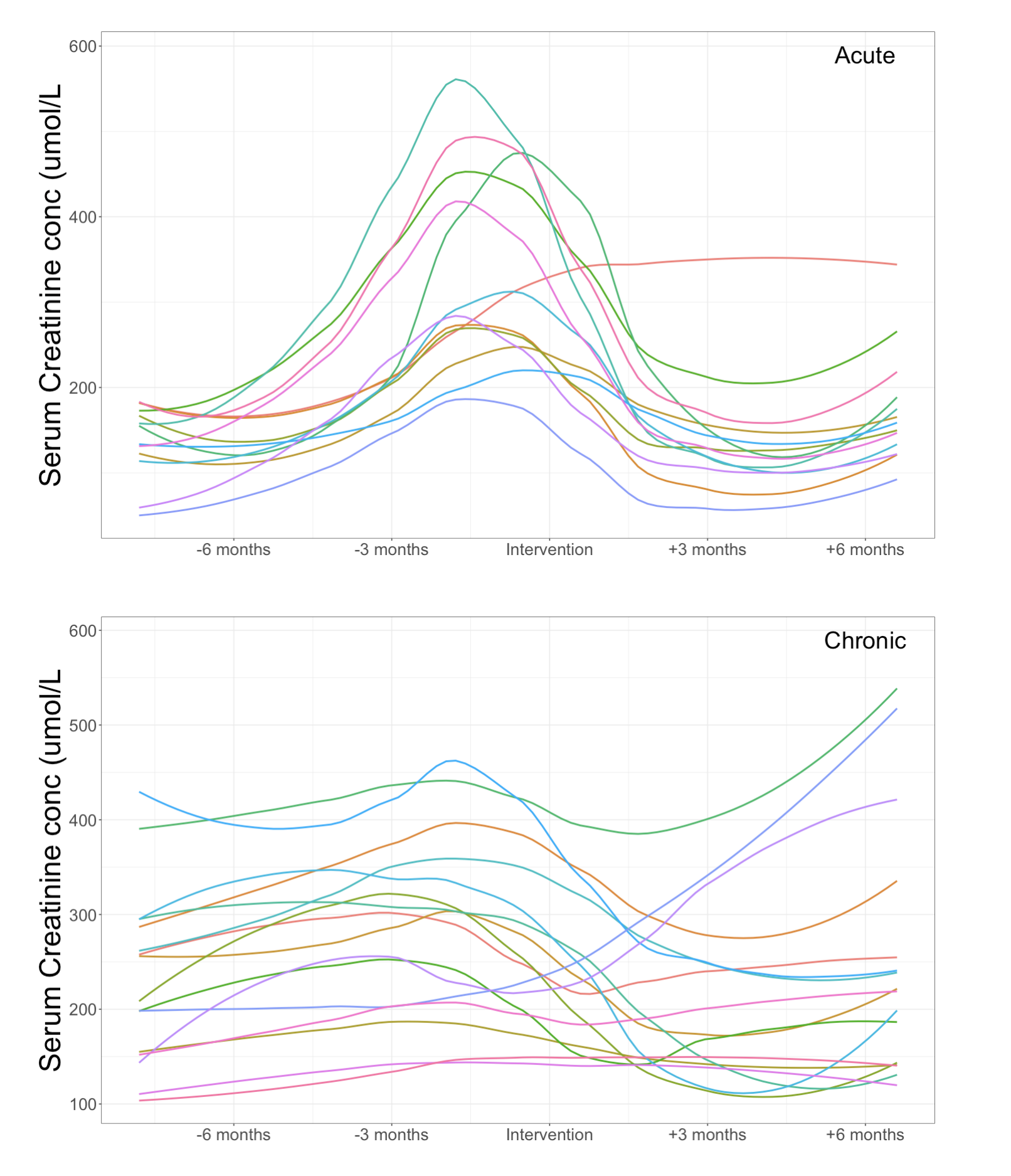

Supplement: Supplementary file 5 — Figure S4 [file 41371_2022_785_MOESM5_ESM.tif]
