# Supplementary material for: The impact of renal artery stenting on therapeutic aims
Source: J Hum Hypertens. 2022 Dec 16;37(4):265–72. doi: 10.1038/s41371-022-00785-8 (PMC10063438; doi:10.1038/s41371-022-00785-8)
Supplement: Supplementary file 6 — Table S1 [file 41371_2022_785_MOESM6_ESM.docx]

|  | | ***Diastolic Blood Pressure*** | | | | | |
| --- | --- | --- | --- | --- | --- | --- | --- |
|  | | ***Baseline*** | | ***Discharge*** | | ***1 Year*** | |
|  | | *Mean* | *+/-* | *Mean* | *+/- p-value* | *Mean* | *+/- p-value* |
|  | |  |  |  |  |  |  |
| ***Overall***  n = 74 | | *88.7* | *19.1* | *76.8* | *12.7 <0.0001* | *78.5* | *14.1 0.001* |
|  | | | | | | | |
| **Indication** | |  | | | | | |
| ***Hypertension***  n = 51 | | *93.6* | *20.2* | *77.3* | *14.3 <0.0001* | *79* | *16.2 0.0007* |
|  | |  |  |  |  |  |  |
| ***Renal Dysfunction***  n = 29 | | *84.3* | *15.4* | *77.5* | *10.5 0.07* | *77* | *11 0.07* |
|  | |  |  |  |  |  |  |
| ***Pulmonary Oedema***  n = 30 | | *81.8* | *13.5* | *76.4* | *10.4 0.09* | *78.1* | *11.6 0.28* |
|  | | | | | | | |
| ***CORAL*** | |  | | | | | |
|  | *Inclusion*  n = 52 | *88.6* | *20.6* | *76.1* | *13.6 0.0005* | *77.9* | *15 0.004* |
|  |  |  |  |  |  |  |  |
|  | *Exclusion*  n = 22 | *88.7* | *11.7* | *79.7* | *8.7 0.03* | *81.1* | *10.3 0.1* |
